# Editorial: Evolutionary mechanisms of infectious diseases, volume II

**DOI:** 10.3389/fmicb.2023.1192566

**Published:** 2023-04-03

**Authors:** Zhan Zhou, Jianying Gu, Yufeng Wang

**Affiliations:** ^1^Institute of Drug Metabolism and Pharmaceutical Analysis, College of Pharmaceutical Sciences, Zhejiang University, Hangzhou, Zhejiang, China; ^2^Department of Biology, College of Staten Island, City University of New York, Staten Island, New York, NY, United States; ^3^Department of Molecular Microbiology Immunology, South Texas Center for Emerging Infectious Diseases, University of Texas at San Antonio, San Antonio, TX, United States

**Keywords:** evolution, infectious diseases, infection, pathogenicity, virulence

Infectious diseases continue to pose significant global health challenges, as they are among the leading causes of mortality worldwide, according to the World Health Organization (WHO) (WHO, 2020). The ongoing emergence and re-emergence of infectious pathogens highlight the complexity and dynamism of these diseases, underscoring the need for proactive and adaptive approaches to their management and control (Morens et al., 2004; Morse et al., 2012; Morens and Fauci, 2020; Frutos et al., 2021; Baker et al., 2022).

Infectious diseases are dynamic and constantly evolving, driven by a variety of evolutionary mechanisms that enable pathogens to adapt, survive, and spread within host populations. Understanding these mechanisms is crucial for developing effective prevention and control strategies. Recent advances in the study of “evolutionary mechanisms of infectious diseases” have seen significant progress (Woolhouse et al., 2005; Sironi et al., 2015; Geoghegan and Holmes, 2018; Gomez-Carballa et al., 2020; Seitz et al., 2020; Cao et al., 2022; Obermeyer et al., 2022). Scientists have unraveled the complex interactions between pathogen evolution, antibiotic resistance, and host adaptation through in-depth analysis of pathogen genomes, with the help of high throughput omics technologies and big data analytical approaches (Didelot et al., 2016; Grote and Earl, 2022; Zhou et al., 2023). Pathogens rapidly adapt to environmental changes *via* mechanisms such as mutation, genetic recombination, and horizontal gene transfer (Frost et al., 2005; Shi et al., 2022). Furthermore, research on host immune systems and microbiomes helps understand disease transmission and outbreak dynamics (Virgin, 2014; Zheng et al., 2020). These developments have spurred the creation of novel vaccines and antibiotics to combat the growing threat of infectious diseases (Rappuoli and Aderem, 2011; Excler et al., 2021). In the future, interdisciplinary research and collaboration will enhance prediction, prevention, and control of disease transmission (Morse et al., 2012; Zeng et al., 2021).

The second volume of our Research Topic, “*Evolutionary mechanisms of infectious diseases*, volume II," builds on the foundation established by the first volume (Gu et al., 2021), further delving into the complex interplay between pathogens, hosts, and the environment. This collection of articles expands our understanding of the evolutionary processes underlying infectious diseases and highlights the importance of a multidisciplinary approach to tackle the challenges that they present. By studying the evolutionary mechanisms, researchers can gain valuable insights into the processes driving the emergence, spread, and persistence of infectious diseases (Woolhouse et al., 2005; Geoghegan and Holmes, 2018). This knowledge can inform the development of more effective prevention and control strategies, such as targeted vaccination campaigns (Andre et al., 2008; Kuehn, 2022), antimicrobial stewardship programs (Dyar et al., 2017; Rice, 2018), and surveillance systems to monitor and respond to emerging and re-emerging pathogens (Morse et al., 2012; Baker et al., 2022).

We sincerely thank all contributors of our Research Topic. This collection of 11 articles is divided into four sections. The first section includes four articles presenting comprehensive genomic analyses of viral and bacterial pathogens, such as severe acute respiratory syndrome coronavirus 2 (SARS-CoV-2), avian influenza viruses, classical swine fever viruses, and Mobiluncus, offering insights into their evolution, transmission dynamics, and interactions with host immune systems. (1) Sun Q. et al. explore the impact of synonymous mutations on the SARS-CoV-2 genome, revealing their potential functional correlations and providing valuable insights for better pandemic control by analyzing the synonymous evolutionary rate and its relationship with host proteins. (2) Liu, T. et al. present long-term surveillance of avian influenza viruses (AIVs) in the live bird market in Shandong province, identifying H9 as the predominant subtype and providing insights into the epidemic and evolution of AIVs, which can inform more effective control strategies in China. (3) Liu Y. et al. reanalyze 203 complete genomic sequences of classical swine fever viruses (CSFVs), identifying new lineages and potential natural recombination events involving vaccine and highly virulent strains, emphasizing the need for careful vaccine applications and alternative preventive strategies for better CSFV management. (4) Li Y. et al. present the first genome-level analysis of *Mobiluncus*, a pathogen linked to bacterial vaginosis, uncovering phylogenetic distinctions, functional diversification, and evolutionary dynamics that could contribute to better understanding and treatment of the infection.

The second section presents in-depth investigations into the role of non-coding RNAs, such as circular RNAs and long non-coding RNAs, in the regulation of viral replication, host immunity, and the development of novel therapeutic targets. Sun Y-S. et al. compare the transcriptome profiles of mRNA and lncRNAs in human lung epithelial cells infected with different SARS-CoV-2 strains, identifying differentially expressed genes and pathways that may explain the varying replication and immunogenicity properties of the strains, thus offering insights into the pathogenesis of SARS-CoV-2 variants. Liu, T. et al. reveal that circDDX17, a circular RNA, promotes Coxsackievirus B3 (CVB3) replication and regulates NOTCH2 by acting as a miRNA sponge for miR-1248, offering new insights into the role of non-coding RNAs in viral infections.

The third section is composed by four articles focused on the impact of mutations, recombination events, and other genetic factors on pathogen evolution, emphasizing the importance of understanding these mechanisms to inform the development of effective prevention and control strategies. (1) Gao and Zhu investigate the origin of ACE2 binding in sarbecoviruses, a group of evolutionarily related β-coronaviruses including SARS-CoV-2, suggesting that three distinct ancestral RBDs independently developed the ACE2 binding trait through parallel mutations, providing insights into the mutation-driven evolution of sarbecoviruses in their early history. (2) Li F. et al. reveal the molecular evolution, diversity, and host tropisms of Foot-and-Mouth Disease Virus (FMDV) Serotype O in Asia, finding that the Cathay topotype has evolved at a higher rate and displayed increased genetic diversity, becoming a more severe epidemic in recent years, with a distinct host preference compared to other topotypes. (3) Mizzi et al. investigate genetic diversity of *Mycobacterium avium* subspecies, finding that unique coding sequences and mutation hotspots may serve as biomarkers for understanding virulence mechanisms and host/tissue specificity, which could lead to new diagnostic targets and advances in epidemiology and therapeutics. (4) Liu Z. et al. identify novel lineage-specific large sequence polymorphisms in *Mycobacterium tuberculosis* complex, providing insights into the genealogical differentiation and aiding in the development of stable genetic markers for genotyping.

The last section of the collection includes investigations into the complex interplay between pathogens and host immune responses, highlighting the need for a comprehensive understanding of host-pathogen interactions in the context of infectious disease evolution and control. Li J. et al. reveal that the vgrG2 gene in *Burkholderia thailandensis* plays a critical role in its pathogenicity, interaction with host cells, and host immune response, providing new insights into the bacterium's virulence mechanisms.

The articles featured in “*Evolutionary mechanisms of infectious diseases, volume II*” showcase the power of a multidisciplinary approach in deepening our understanding of infectious disease dynamics. As we continue to confront the challenges posed by emerging and re-emerging pathogens, fostering collaboration and innovation across disciplines is more critical than ever.

We extend our heartfelt gratitude to all the authors who contributed their valuable research to this topic, and to the reviewers for their diligent and insightful evaluations. We hope that this collection of articles will serve as a valuable resource for researchers, inspire further scientific inquiry, and contribute to the global effort to combat infectious diseases.

## Author contributions

All authors listed have made a substantial, direct, and intellectual contribution to the work and approved it for publication.
